# Hospital and Institutional News

**Published:** 1915-11-27

**Authors:** 


### HOSPITAL AND INSTITUTIONAL NEWS.

### OUR NOVEL CHRISTMAS NUMBER.

The predominant note in relation to all businesses and public institutions, and also in responsibly managed families, owing to the war, is economy. The deeper and more exhaustive the knowledge of the facts, the greater the practical apprehension of the actual circumstances which dominate the presence or absence of economy in households, in institutions, in business undertakings, the more fully is it realised that an absence of practical training and knowledge is the most fruitful source of waste and extravagance. The circumstances and sorrows of the year 1915 make increasing numbers of people feel reluctant to entertain the idea this year of Christmas festivities and entertainments. The question of a Christmas Number has, therefore, presented special difficulties, with the result that we propose to publish a novel Christmas Number which cannot fail to be helpful to the heads of households, institutions, and great businesses. The aim will be to direct the thoughts of the reader to channels and facts which should prove fruitful in results by enabling economy to be practised readily and with knowledge. Christmas will receive due attention, but our main themes will be practical and suggestive. The modern hospital is a marvellous development, most complicated in its ramifications, remarkable for the evidence it affords of immense growth in science and practice, whilst it yields much living interest by its effects upon the maintenance of the mental and bodily vigour of His Majesty's lieges.

### READERS! LEND A HAND.

We shall attempt to demonstrate all this, to secure the interest and add to the clearness of each subject by illustrations. It may be news to hear that one-fifth of the whole expenditure in a modern hospital goes to the provision of textiles in the fullest meaning of the term. Their quality and adequate supply, as we propose to show, are essential factors to success in the domestic department of a great hospital. At no time probably have drugs and all matters which relate to treatment, cost as much as they do to-day, and an article on galenicals will point the way to saving and economy, with increased efficiency. The greater the ability and efficiency of the administration of a modern hospital, the surer is the just pride taken in its up-to-date equipment. We hope to point the way to important developments which should bring more reputation and increased money to the voluntary hospitals. The year 1915 has produced many novelties, and amongst the most striking and suggestive of these may properly be placed the magazine of the military hospital, originating with the orderlies, many of whom are men of high intellectual power and great artistic ability. It will be our privilege to show how such a magazine can be conducted with success and prove fruitful in results. Why should not 1916 give many an enterprising voluntary hospital its hospital magazine? We are open to suggestions and shall welcome well and tersely written copy regarding notable incidents in connection with our wounded warriors. Also illustrations typical of the year and its special work. 1915 has proved an arduous year for the Editor, and he invites his readers to join hands with him in making this novel Christmas Number of historical value, and memorable as a spontaneous co-operative production.

### THE INEVITABLE HAPPENS.

The committee of the Norfolk and Norwich Hospital have met to appoint a successor to Mr. Hazell. They selected one gentleman from amongst the candidates for election, but he declined the appointment. In the result no one was elected to fill the vacant secretaryship. Mr. Percy L. Vaughan, a member of the Council of the Incorporated Association of Hospital Officers, who has been assistant secretary at St. Luke's Hospital, London, for nine and a half years, with the consent of his committee, is acting as *locum tenens* at Norwich for three months.

### THE BRITISH HOSPITALS ASSOCIATION.

Mr. H. Wade Deacon, chairman of the Royal Infirmary, Liverpool, who is also chairman of the Council, made an interesting speech at the annual meeting on the 18th instant (see page 189). He put forward very high claims for the work done by the Council in the absence of a conference during 1915. One was that all voluntary hospitals who would accept payment now received grants from the Government to defray the cost of wounded sailors and soldiers admitted to treatment as in-patients. He was of opinion that all hospital employees of military age should offer themselves as recruits under Lord Derby's scheme, when the hospitals where they were employed could, in the case of those whose services were indispensable, apply to the proper authorities for their release from enlistment. Mr. Deacon has rendered yeoman service of great value to the voluntary hospital system in Liverpool, where he is held in honour and universally trusted. The election of Mr. Courtney Buchanan, of the Metropolitan Hospital, as a joint honorary secretary should strengthen the work of the Association.

### SOME POINTS WORTHY OF ATTENTION.

We are informed there is a widespread feeling outside the Council of the British Hospitals Association among the provincial voluntary hospitals that the latter should be fully represented on the executive staff. There is much work of a useful kind which the Association under active administration might with advantage undertake. This end, it is thought, would be furthered by following the usual course of similar societies and appointing a small committee of active and knowledgeable hospital men to arrange subjects for papers and prepare each year a programme for the winter and spring sessions. This committee could then secure speakers with knowledge to take part in the discussions, and so put more life and usefulness into the organisation. In this way new members would be obtained and the Association's greater usefulness assured. The Association is indebted to Mr. Sydney A. Smith, F.S.I., for a highly technical and exhaustive paper on the subject of "Provisional Valuations under the Finance Act." He pointed out that in London various hospitals are collectively paying over £25,000 a year in rates—a sum which, though important to the hospitals, is too infinitesimal to be felt in any sense whatever by the ratepayers. In fact, £25,000 represents but half a farthing in the £ for the whole of London. This paper will presumably be published in due course with the proceedings of the Association for the year. It belongs to a type which primarily affects the domestic affairs of the hospitals, and it would be well to make a distinction between this useful class of papers and those of wider interest to the public. Such a reform would remove any risk of damage to the hospitals by attracting publicity to matters whereby harm, rather than good, might ensue to the institutions most concerned. This is not the time to raise discussions upon questions which are happily asleep. If left alone, such questions after the war are not likely to be revived, and there is good ground for hoping the hospitals may not be troubled further with them. The hope was expressed that the next conference, when circumstances are favourable, may be held at Liverpool.

### SHOULD A HOSPITAL BUILD IN WAR TIME?

This question, as a matter of policy, was raised at a special meeting of the governors of the Herefordshire General Hospital lately held to decide whether or not to proceed with the building of a Nurses' Home, which is generally admitted to be badly wanted. As a result of a discussion last July, when Mr. J. A. T. Nicholson laid plans before the meeting, a committee was appointed to enter into details, and to consider the desirability of retaining the hospital's private nursing staff. The report of this committee, considered at the special meeting referred to, recommended the retention of the private nursing staff, and the acceptance of the plans provided by Messrs. Nicholson and Clarke at an approximate cost of £4,000. The report concluded with these words: "The matter to be proceeded with in its entirety when considered advisable." This, of course, begged the general question as to whether building should be commenced at once; after discussion it was decided to postpone the carrying out of the scheme. Towards the cost, which might exceed £4,000, £2,000 is in hand. Since one speaker declared that the existing Home has been condemned on more than one occasion already, we do not think the public should refuse to support the scheme. There can be no hard-and-fast rule against building, even at the present time, if two conditions are fulfilled. The first is that there can be no question about the urgency of the proposed addition; and the second that there is a reasonable prospect of finding the necessary money.

### A TYPICAL GERMAN SLANDER.

We are very glad that the Secretary of the Admiralty has promptly nailed to the counter the malignant German slander that British hospital ships are used for the transport of troops, munitions, and other war material. The accusation was first made as a "conjecture" in the German official wireless news, followed a few days later by an assertion that "sworn statements in German hands give proof" of this childish slander. The charge comes with peculiar grace from a nation whose maritime methods of war have outraged not merely every letter of international law, but also every canon of humanity. It is branded by the Secretary of the Admiralty as "absolutely false"; the contradiction was not needed for the citizens of any nation whose people go down to the sea in ships, but there may be some which know little of the proud traditions of the British Navy, and for them the denial may not be superfluous.

### THE SOUTH COAST MILITARY HOSPITALS.

Interesting announcements are to hand concerning the War Office scheme for eight large military hospitals on the South Coast. It is expected that no fewer than 30,000 beds are to be provided, and sites have been inspected at Bognor, Littlehampton, and Worthing. In the case of Worthing, Mr. Scott, the chief sanitary inspector of the Eastern Command, has been in touch with the Worthing authorities, where certainly one, and probably two, military hospitals will be established. Each of these institutions is to accommodate 4,000 soldiers, and it is hoped that they will be ready for the reception of patients in the beginning of the New Year. The object of the scheme as a whole appears to be to reduce the number of wounded in France by transferring them to hospitals in this country. The scheme is a big one, and its progress will be watched with interest particularly in the seaside towns concerned, who may, perhaps, through the presence of the new institutions, begin to make good some of the losses they have experienced from the absence of their usual visitors.

### WORKERS AND THE MAINTENANCE OF INFIRMARIES.

At the last meeting of the Oldham Trades and Labour Council, the Amalgamated Society of Engineers brought forward a resolution suggesting that the local Royal Infirmary should be taken over by the municipality with the aid of the out-townships, and should be rate-supported. The view of the engineers' trade union was that the infirmary should not have to rely upon voluntary effort for its support, and the remark was made that it was a disgrace to the town that the institution should be worked with a shortage of funds. The remedy for the unfortunate state of affairs which called forth this resolution is plain, and Mr. Critchley, the representative of the Council on the Infirmary Committee, touched the key to the whole matter when he declared that the collections for the infirmary were not supported as they ought to be by the workers. For his own part, he said, he would not object to a compulsory levy on the workers for the upkeep of the institution. That, however, is quite unnecessary if only the workers will realise their responsibilities. When one comes to consider the immense wealth of some of the Lancashire trade unions, and the excellent earnings of mill and workshop operatives—at any rate in peace time—it is a matter of some difficulty to understand why the claims of the local hospitals do not receive more generous consideration. The resolution of the Amalgamated Society of Engineers was withdrawn, and it is earnestly to be hoped that the working classes of Oldham will see to it that the institution receives the support it merits.

### DR. BREND AND THE LIMOGES HOSPITAL.

We regret that a paragraph in our last issue should have been interpreted as meaning that Dr. W. A. Brend is still connected with the administration of the National Insurance Act. This is incorrect, as Dr. Brend two years ago resigned the appointment which he formerly held. We regret the misconception which appears to have arisen, and welcome Dr. Brend to the field of administrative medicine, where his high qualifications should prove of great practical value.

### POOR-LAW OFFICIALS AND ENLISTMENT.

Poor-Law officials of military age all over the country are enlisting in the Navy and Army. This is causing a great deal of embarrassment to Boards of Guardians, who are, however, in the great majority of cases, meeting the difficulties in a statesmanlike spirit, and giving their officials every encouragement in their desire to serve their country. It is not at all easy, however, to find substitutes for all the men and women who are thus leaving the Poor-Law Service for the duration of the war. Naturally, it is the skilled workers—the medical men, nurses, engineers, relieving officers, etc., who are most difficult to replace. There is here an inviting opportunity for superannuated Poor-Law officials to take part in the national work. Many of them are still capable of doing useful service, and wherever possible they should come forward now, so as to release the more active and vigorous members of their old profession.

### POOR-LAW AMALGAMATION IN SHROPSHIRE.

The Atcham Board of Guardians have decided to ask the Local Government Board to take the necessary steps to amalgamate the Unions of Atcham and Church Stretton. The proposal is an excellent one, and the Local Government Board, in the interests of economy and efficiency, should give effect to it. Atcham is a large Union, with a population of over 51,000, whilst Church Stretton has a scattered population of only 6,300. It is impossible for a Union so small as this to deal efficiently with its sick poor. By amalgamation Church Stretton would gain the advantage of the use of the better-equipped institutions possessed by the Atcham Guardians. The economy which the war expenditure will force upon the nation renders concentration of effort essential if the poor are not to suffer unduly. The Church Stretton Guardians are opposed to the amalgamation, but their objections to absorption in the neighbouring Union should not be allowed to stand in the way of a reform the advantages of which are obvious to any unprejudiced outsider.

### NEW MEDICAL BENEFIT REGULATIONS.

The Insurance Commissioners have issued a draft of revised medical benefit regulations which will take effect from the beginning of 1916. The main object of this revision is to give effect to the recommendations of the Departmental Committee on the Drug Tariff (The Hospital, September 25, p. 542). Had these recommendations been adopted in full, the chemists would have had the first claim on the Medical Benefit Fund, and there would have been some risk of an encroachment on the seven shillings allotted to Insurance practitioners. This proposal, however, was unanimously rejected by Panel Committees, and the Commissioners have been reluctantly compelled to give way to the doctors and to proceed with the enforcement of the new tariff without disturbing in any material way the position of the doctors. The Treasury has undertaken to guarantee that under the new conditions the chemists' accounts shall be paid in full, and thus the difficulty has been overcome. But it is doubtful whether, under the new tariff, there will be any shortage, and whether there will be any necessity to have recourse to the public funds; for the amount of the accounts will probably be reduced by something like 20 per cent., and the balances of the Drug Fund remaining in each area after the bills have been paid and the doctor has received his "floating" sixpence, are to be pooled and utilised for paying the chemists' accounts in those districts in which there is a shortage. Panel practitioners are to be congratulated on their successful opposition to the Commissioners' original proposals. Any attempt to encroach on the amount of the fees agreed upon when the Insurance Act came into operation should be defeated at all costs.

### AN IMPORTANT CASE.

A case of some considerable importance to National Health Insurance practitioners has recently been heard by Mr. Justice Rowlatt in the King's Bench Division. The plaintiff was Mr. C. W. Moore, M.R.C.S., L.R.C.P., who was until recently a doctor on the Leicester Panel, and he sought an injunction to restrain the Leicester Insurance Committee from surcharging him £31 7s. in respect of alleged over-prescribing in 1913. After inquiry, in the manner which the Insurance Committee presumably considered to be in accordance with the regulations, the sum in question was deducted from Mr. Moore's account. It was argued by plaintiff's counsel that the Commissioners had no power to make regulations to take away from the doctor that which he was given by the terms of the Act and his agreement with the Insurance Committee. Judgment was given for the plaintiff, with costs. If it is ultimately established that the Commissioners' regulations in respect of the surcharge for over-prescribing are *ultra vires*, there will no doubt be many other claims for the refund of money that has been withheld from doctors similarly placed to the plaintiff in this case. Under the new regulations which will come into force in January next, however, the responsibility of controlling extravagant prescribing will be thrown upon local Panel Committees.

### A NICE POINT.

In The Hospital of the 6th inst., p. 118, attention was drawn to some of the disadvantages attaching to the system of election of applicants for charity by subscribers' votes; and earlier, in reference to the Putney Hospital for Incurables, where that system prevails. A correspondent now puts before us a slightly different point from any then discussed, and one that raises a very debatable question. Our reader complains that she was canvassed by friends to give some votes to a certain man in whose case their support had been enlisted; a printed card, issued on behalf of the patient and signed by a doctor and a clergyman, amongst others, described his illness as "paralysis." On receipt of the official list of candidates from the secretary of the Putney Hospital, it was seen that the diagnosis there given was locomotor ataxia. Our correspondent had taken some trouble to enlist further supporters for the man, and felt aggrieved when she discovered that his disease was one incurred through his own misconduct in the past. She does not contend that this should debar him from help and sympathy now, but only that his relief by this charity can only be achieved by the defeat of some other candidate whose plight may be equally sad, and to whom no suggestion of reproach attaches. We agree with her that the card sent round on behalf of the patient contains a *suppressio veri* which should not have been countenanced by a doctor or by a priest; the other point as to whether disseminated sclerosis (for example) should receive priority over general paralysis, locomotor ataxia, and diseases of similar origin, at the hands of a public charity is a nice one, and deserves fuller discussion.

### THE GLASGOW WOMEN'S PRIVATE HOSPITAL.

About a year ago an institution known as the Glasgow Women's Private Hospital was opened at West Cumberland Street. Its intention was to supply adequate treatment for women of limited means, and for women who cannot be suitably treated in their own homes. In its first year a move was made to larger premises, at 11 Lynedoch Place, where, it should be added, the patients are attended by women doctors. In the new house there are two wards with five beds in each, besides a few private rooms. The patients pay fees, but these are estimated only to defray half the expenditure, and the success of an appeal issued in June for funds to pay the cost of removal leads the committee and the hon. secretary, Miss Sylvia Murray, to hope that the enlarged institution may be maintained. The movement in favour of such institutions as the Florence Nightingale Hospital for Gentlewomen, and for pay wards in connection with the voluntary hospitals, is one which may probably receive an impetus after the war from the demand there is likely to be from many straitened homes. But is the policy wise of restricting medical attendance to that of women doctors?

### THE WOMEN'S PAY HOSPITAL AT DUNDEE.

The first report available since the Dundee Women's Hospital and Nursing Home moved into its new enlarged building in February is of interest to all who follow the progress of what may be called the pay-ward movement. The number of patients received during the year has been 136, with a daily average of twelve, and the patients are those who wish to pay their way in time of sickness while having advantageous surroundings unobtainable in their own homes. It is interesting to note that the patients' payments amounted to £564, while the subscriptions were £181. The latter sum does not seem very large, but though the expenditure amounted to £1,273, leaving a deficit of £441, this debt is due to the cost of furnishing the hospital and the provision of surgical appliances, so that it may be regarded as capital expenditure which will not recur. There is an endowment fund of £2,385, and also a bazaar fund of £1,946. In these circumstances the new institution seems to have started on a useful career, and to be equipped upon a satisfactory financial basis.

### THE RIGHTS OF ASYLUM ATTENDANTS.

A curious case has just been decided in the King's Bench Division in Dublin in favour of John Carroll, an asylum attendant, and against the Armagh District Lunatic Asylum authorities. Carroll had completed the necessary service entitling him, if permanently disabled, to a pension at a certain scale under a superannuation scheme. He applied for pension on the ground of incapacity for further service, and produced three medical certificates of such incapacity. The asylum authorities had him inspected by some of their own doctors, who reported that the man was not permanently incapacitated for his duties, nor indeed incapacitated at all. Carroll thereupon exercised a statutory right of laying his case for adjudication before the Lord Lieutenant, who decided in his favour on the strength of the medical certificates he submitted. The asylum managers had no opportunity of laying their version before the Lord Lieutenant (Lord Aberdeen), and contended that the latter had no right to dispose of the case without proper inquiry. The Court took the view that they had no power to go behind the Lord Lieutenant's decision. It would appear on the face of it that the latter's action was precipitate, and in a country where political corruption is as rife as it is in Ireland the statute certainly opens up very undesirable possibilities of intrigue.

### THE LONDON JEWISH HOSPITAL.

The projected Jewish Hospital has got beyond the stage of incubation, and has entered that of invasion; for its foundation-stone was laid on November 14 at Stepney Green by Mrs. Flora Solomon D. Sassoon. It is stated that the site has cost £5,000, and that the building will require another £33,000; the former is paid for, and £13,000 is already in hand towards the latter. The out-patient department, which will be open to Gentiles as well as Jews, will cost about £6,000, and will be completed early next year. The hospital will contain 120 beds, and will be the second of its kind in England; Manchester has already taken priority of London in this respect. The President (Mr. I. Berliner) explained at the ceremony that the new hospital is in no way to be regarded as a criticism of, or in opposition to, existing institutions, where Jews are well treated; but it is felt that their own people require a hospital where they would find no difficulties in the matters of language and sentiment.

### DEATH OF DR. BASTIAN.

The death of Dr. H. Carlton Bastian at the age of seventy-eight removes not only a well-known hospital physician of very strongly marked personality but one who was in his day a protagonist of some still-debated scientific theories and a man of immense energy and versatility. He was for many years physician to University College Hospital and to the National Hospital to the Paralysed and Epileptic, Queen Square; on his retirement from active service he became consulting physician to both those institutions. He was also Professor of Medicine at University College. Not content with eminence in neurology and in general medicine, Dr. Bastian took a great interest in biology, and was the chief exponent of the hypothesis of abiogenesis, or the origin of life independently of pre-existing life. As long ago as 1870 he was hotly engaged in controversy with Darwin, Huxley, Tyndall, and Pasteur on this subject; and to this day he had been unable to obtain acceptance for his views, though he continued undauntedly to advance them. In the felicitous phrase of the *Times*, Dr. Bastian "knew no compromise, sought no discharge"; he was a born fighter, and even if his peculiar views are eventually proved to be wrong, he rendered many services both to science and to neurology. Last June he was granted a Civil List Pension in recognition of those services.

### WORKING MEN'S SUPPORT IN THE MIDLANDS.

In placing before the Wolverhampton Hospital Committee the need for securing further support to the general hospital of the town, Mr. W. H. Harper, the hospital's secretary, gave his audience some instructive figures as to the measure of working men's support to various hospitals in the Midlands. After pointing out that the general hospital was threatened with a growing deficit, now likely to amount to £2,000 on the current year, Mr. Harper reminded the Saturday Committee that they provided 39 per cent. of the total income of the hospital. He gave figures to show that at Coventry the workmen provided 50½ per cent., at West Bromwich 54 per cent., and at North Stafford 66 per cent. He might have added further instances, but the above was sufficient for his purpose—namely, to show that the workmen at Wolverhampton were some way down the list. The Saturday Committee is to report to-day (27th), after consultation with workmen, as to the latter's decision with regard to raising their subscriptions. The Midland towns have proved such strongholds of working men's support of hospitals that it can hardly be doubted that the Saturday Committee will be instructed to guarantee a special increase on the subscriptions of those they represent.

### EXTORTION AND CHARITY FETES.

Correspondents in the Irish Press are discussing, or rather criticising, a method adopted by the organisers of matinées for charitable purposes which consists, it seems, in the distribution of tickets to those whom the organisers consider would not like to return them. As one of the secretaries of a patriotic effort in Belfast has written to defend the practice, it is worth while examining the grounds on which he does so. Alas! all he has to say is that grumblers are not those by whom funds are raised, which, of course, is irrelevant to the question of the method of raising them; although he finally adds the saving clause that "those who cannot afford to contribute are not expected to do so." This cannot be supported by acceptable evidence. It is not a question of theory, but one of practice, as to where the line between persuasion and compulsion should be drawn. A line there is, which there is an inevitable tendency nowadays to overstep. The method of nominally giving articles away—like matinée tickets—and then collecting the price of them, as presumably is being done in Belfast, rests upon practices which are sharp rather than honest. To say this is to condemn them. The plan can find no place where decent people will suffer it.

### INDUSTRIAL SCHOOLS VERSUS HOME LIFE.

In the recently published Report of the Chief Inspector of Reformatory and Industrial Schools, Mr. C. E. B. Russell, considerable emphasis is laid upon the value of home life to the growing child. With all the advantages that a residential school possesses there is, in the opinion of the Inspector, some risk of the boy or girl becoming selfish and unfitted to face those great emergencies of life which are only too familiar to the children of the poorer sections of the community. Mr. Russell, therefore, urges managers and superintendents of this class of school to adopt a policy of early licensing—that is, the returning of a boy or girl on licence to his or her home or to suitable employment away from home after eighteen months in a school. He also advocates consultations with suitable parents as to the disposal of their children on leaving the school. He further looks forward to an extended use of the power given to school managers to board out little children under eight years of age, and he trusts that when once boarded out the children will not at a later date be transferred to industrial schools without adequate cause and serious consideration. We are all indebted to the State Children's Association for their wholehearted support of the Chief Inspector's recommendations in the interests of the children.

### RANJI'S GIFT TO LEEDS INFIRMARY.

Among the Indian magnates who have already helped our wounded by providing buildings for their treatment and funds for maintenance, Prince Ranjitsinhji's name has received honourable mention. He has converted a country house into a hospital, been a generous subscriber, and now has made a special gift to the General Infirmary at Leeds. This is to take the form of an annual gift of one hundred guineas on his birthday, in recognition of the successful treatment which he experienced in a Leeds nursing home, where he had gone as a patient when suffering from a shooting accident involving an injury to his right eye. The reason for the gift is to be perpetuated by the maintenance of a new ophthalmic out-patients' department at the infirmary. Prince Ranjitsinhji, whose repute as a cricketer in this country has overshadowed his official position as the Jam Sahib of Nawanagar, is reported to have left England for India, to attend his sister's marriage to the Maharajah of Jodhpur.

### NOTTINGHAM SATURDAY FUND AND THE WOUNDED.

A bold statement was made by Mr. C. H. Crofts, and endorsed by the chairman, at a recent meeting of the Hospital Saturday Committee at Nottingham. It was emphasised that in spite of the number of wounded received at the general hospital "the accommodation for civilians had not been diminished." The chairman, Mr. James Forman, laid stress on this point owing to the suggestion contained in a workman's letter to the effect that the admission of civilians was delayed on account of the soldier patients. The matter is important since Mr. Crofts has urged that the committee ought to endeavour to maintain at least another hundred beds. To do this, the fund's contribution would have to be increased to £15,000, a large sum which could be forthcoming only by the united effort of all whom the Saturday Fund Committee represents. The need for supplying hospital beds to the wounded is declared so urgent, however, that the hospital managers did not hesitate to admit some curtailment of the facilities offered to civilians. Very few hospitals indeed would be in a position to declare that these facilities are wholly unimpaired.

### MORE ACCOMMODATION AT EDMONTON FOR WOUNDED.

The committee of the Edmonton Military Hospital have met officials of the Local Government Board with regard to providing extended accommodation for the treatment of wounded soldiers. It has been decided to make use of the old infirmary, and, if the accommodation which will remain available for the sick poor during the military occupation should prove insufficient, the Guardians will be prepared to make such further arrangements in regard to Poor-Law cases as may be required. The Army Council has expressed its cordial thanks and appreciation of the patriotic spirit of the Guardians in making these further arrangements.

### POPLAR HOSPITAL'S DIAMOND JUBILEE.

It would do good, and prove fruitful in results, if all the men and women who are millionaires, or who have larger means than they can conveniently spend, could make an occasion to visit this hospital and to spend one hour there with a view to enable them to realise the actual number of accidents which pass through its casualty department every day in the year It is war-time, so the annual festival dinner has gone by the board. With a readiness, however, worthy of imitation, the want will be supplied through the kindly interest of the proprietors of the Baltic Exchange, which has enabled the Merchants' Hall of the Baltic to be occupied at five o'clock on December 15 for an hour, when the impressive work of this accident hospital will be graphically demonstrated by Lord Inchcape, Lord Knutsford and Mr. William Crooks, M.P. Imitation is the sincerest flattery, and the keener hospital men might learn a wrinkle or two if they availed themselves of the offer of tea and light refreshments at 4.30, and then stayed to support the hospital and its speakers. Every voluntary hospital manager might borrow a happy idea from Poplar by inviting their subscribers and donors to add 25 per cent. to their usual contribution to meet the present increased cost of food and all medical stores.

### THIS WEEK'S DRUG MARKET.

On the whole, business has been rather quieter, but some departments are as active as ever. Thus manufacturers of those chemical products which are required in such large quantities for the treatment of sick and wounded soldiers continue to work at full pressure, and as a consequence of the abnormal demand and the shortage of labour are unable to keep pace with their orders. Makers of bromides, iodides, bismuth preparations, caffeine, morphine, and so on, are so busy that they cannot undertake to supply orders promptly. Atropine is again dearer, and even at the phenomenal price quoted is hardly obtainable even in small quantities. Another alkaloid used in ophthalmic practice, pilocarpine, is also dearer. Among synthetic drugs the only product that seems to show any signs of receding in price is acetyl-salicylic acid, and even in this case the possibility of any pronounced decline is somewhat remote; but the drug is certainly in better supply, although in some cases the quality leaves much to be desired. Phenacetin is again much dearer. The high price of cod-liver oil is firmly maintained, and there is apparently no reason to hope for lower values during the present winter. Quinine has not receded in price to anything like the extent that had been anticipated, but buyers should act with caution.

