# Addendum

**Published:** 1915-12-11

**Authors:** 


ADDENDUM.

With reference to the note in The Hospital of November 27 (p. 178) we are requested to state that the Glasgow Women's Private Hospital has been in existence for thirteen years.

